# Supplementary material for: Broadband quantum-dot frequency-modulated comb laser
Source: Light Sci Appl. 2023 Jul 25;12:182. doi: 10.1038/s41377-023-01225-z (PMC10368713; doi:10.1038/s41377-023-01225-z)
Supplement: Supplementary file 1 — Supplementary Information for: Broadband quantum-dot frequency-modulated comb laser [file 41377_2023_1225_MOESM1_ESM.docx]

**Supplementary Information for:**

**Broadband quantum-dot frequency-modulated comb laser**

Bozhang Dong^1,*,†^, Mario Dumont^2, †^, Osama Terra^1,3^, Heming Wang^1^, Andrew Netherton^2^, and John E. Bowers^1,2,*^

^1^ Institute for Energy Efficiency, University of California, Santa Barbara, CA, USA

^2^ Department of Electrical and Computer Engineering, University of California, Santa Barbara, CA, USA

^3^ Present address: Primary Length and Laser Technology Lab, National Institute of Standards, Giza, Egypt

*Email: bdong@ucsb.edu, bowers@ece.ucsb.edu

^†^These authors contributed equally

1. **ADDITIONAL MEASUREMENTS**

In the DWDM system in which a large number of ring modulators are deployed, the frequency comb laser should offer a large mode spacing to eliminate the crosstalk between each channel. On the other hand, it should be noted that energy efficiency is another issue that must be addressed. In the system that we are currently building up, 20 comb lines within the 3-dB bandwidth strike a perfect balance between laser performance and energy efficiency. For the QD laser presented in the main text, the maximum comb line number within the 3-dB bandwidth that it can deliver is 30, and the corresponding 3-dB bandwidth is 10 nm (1.82 THz), which meets the requirement of our DWDM system. To achieve this maximum bandwidth (Fig. S1a), the gain is biased at 135 mA and the SA is biased at -4.8 V. Compared to the AM pulse width that is on the order of picosecond, the broadband FM comb that allows for sub-picosecond pulse width is beneficial to spectroscopy, remote sensing, and optical frequency synthesis. The shortest FM pulse width that our QD laser delivers is 495 fs (Fig. S1b), which meets the state-of-art of the QD mode-locked laser.


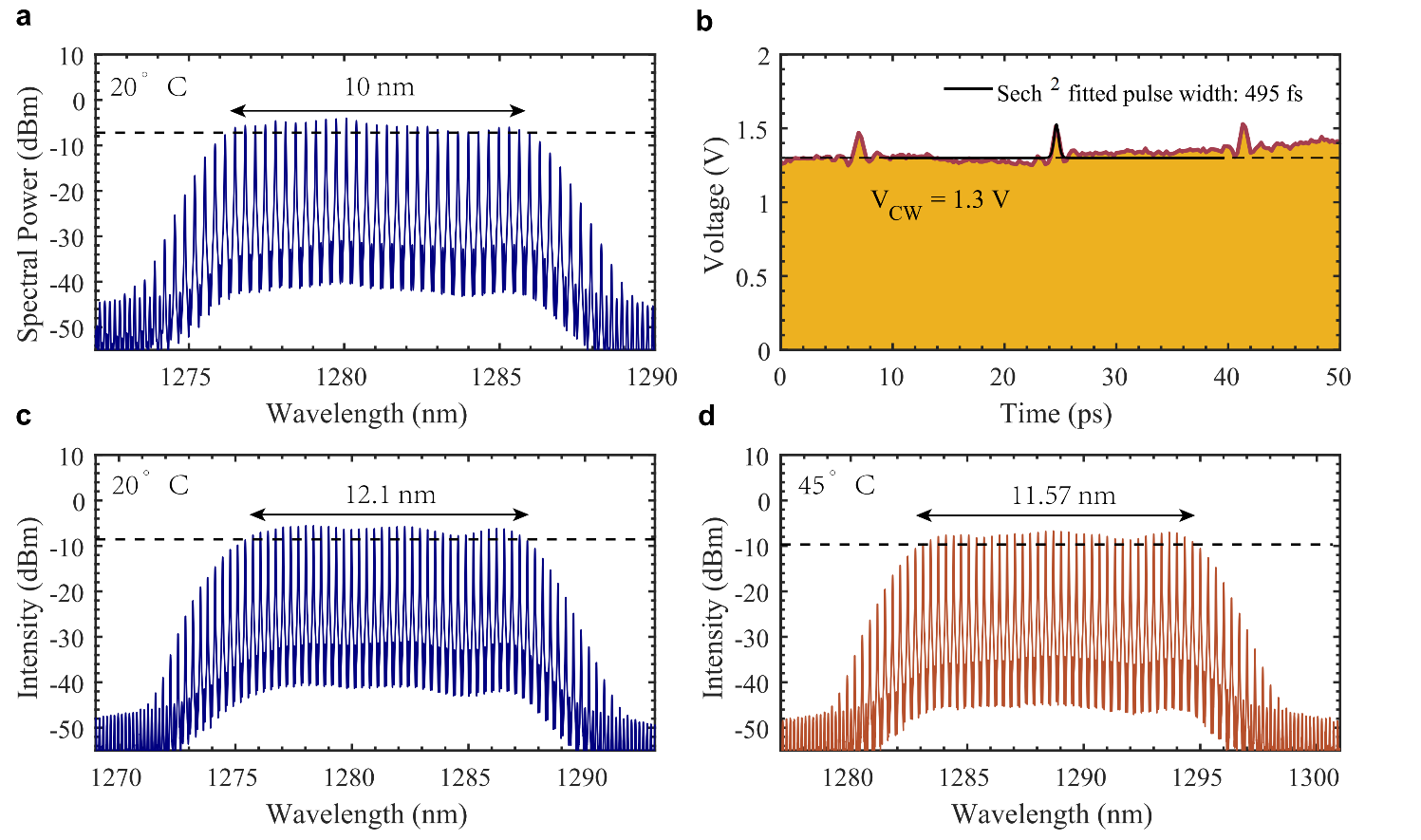


Supplementary Figure 1. **QD FM comb laser. a.** Optimal optical spectrum at 20°C of the QD laser whose SA length is 7.8% of the total cavity length. **b.** Intensity autocorrelation of an FM comb state. Our QD laser delivers short FM pulse width of 495 fs. **c.** Optimal optical spectrum at 20°C of the QD laser whose SA length is 11% of the total cavity length. **d.** Optimal optical spectrum at 45°C of the QD laser whose SA length is 11% of the total cavity length.

For upcoming broadband applications, our QD platform can also deliver a 3-dB bandwidth as large as 12.1 nm (2.2 THz) (Fig. S1c), which is the largest bandwidth that has been reported in the O-band QD laser. This broadband state is achieved by increasing the SA length to 11% of the total cavity length while applying 145 mA on the gain and -4.8 V on the SA. It should be noted that our QD laser does not suffer from an evident reduction in the comb bandwidth when the operation temperature is increased to 45°C. This device still delivers a 3-dB bandwidth as large as 11.57 nm (Fig. S1d), when it operates with 125 mA on the gain and -3.9 V on the SA. However, the increase in the operating temperature results in a redshift of the central comb wavelength from 1281 to 1289 nm.

Performance analysis of the semiconductor mode-locked lasers is summarized in Tab. 1. Compared to the conventional QW lasers, the FM comb generated by the ultrafast QD laser allows for not only a broader optical bandwidth but also a shorter pulse width. Along with the high energy efficiency and high-temperature stability, the QD mode-locked laser will play an important role in the next generation of silicon PICs.

Table 1: Comparison of Mode-Locked Laser on Various Material Platforms and Structures.

| Telecom band | Material platform | Method | Pulse width (ps) | | Repetition rate (GHz) | Optical bandwidth | Operation temperature | Ref. |
| --- | --- | --- | --- | --- | --- | --- | --- | --- |
|  |  |  | AM | FM |  |  |  |  |
| C-band | InGaAsP MQW on Si | Hybrid anti-CPM (external cavity) | 7 |  | 1 | 13 (−10 dB BW) | 20 | [1] |
| C-band | InGaAsP MQW on Si | CPM (external cavity) | 21.2 |  | 10 | 6.4 (−3 dB BW) | 16 | [2] |
| C-band | MQW on Si | CPM (external cavity) | 0.9 |  | 20 | 2.96 (−3 dB BW) |  | [3] |
| C-band | AlGaInAs/InP MQW | CPM (external cavity) | 0.49 |  | 40-240 | 5.47 (-3 dB BW) |  | [4] |
| C-band | InAs/InP QD | Single-section |  | 0.312 | 92 | 11.62 (-3 dB BW) | 18 | [5] |
| C-band | InAs/InP QD | Single-section |  | 0.295 | 10-100 | 17.9 (-3 dB BW) | 18 | [6] |
| C-band | InAs/InP QD | Single-section |  | 0.6 | 34.2 | 11.96 (-6 dB BW) | 18 | [7] |
| C-band | InAs/InP QD | Single-section |  |  | 32.5 | 11.8 (-3 dB BW) | 25 | [8] |
| O-band | InAs/InGaAs on Si | Single-section |  | 0.49 | 31 |  | 20 | [9] |
| O-band | InAs/GaAs QD on Si | Two-section | 5 |  | 20 | 6.1 (−3 dB BW) | 18 | [10] |
| O-band | InAs/GaAs QD | Two-section | 4.9 |  | 25.5 | 4.7 (−6 dB BW) | 20 - 120 | [11] |
| O-band | InAs/GaAs QD | CPM | 0.81 |  | 100 | 11.5 (−3 dB BW) | 25 - 100 | [12] |
| O-band | InAs/GaAs QD on SOI | CPM (external cavity) |  |  | 102 | 6.5 (-3 dB BW) | 25 | [13] |
| O-band | InAs/GaAs QD on SOI | CPM (external cavity) |  |  | 15.5 | 12 (-3 dB BW) | 23 | [14] |
| O-band | InAs/GaAs QD | CPM | 1.7 | 0.495 | 60 | 12.1 (-3 dB BW) | 20 - 45 | This work |

1. **INTERMODE BEATING PHASE MEASUREMENT**

The beating of the external tunable laser (TNL) and the m*^th^* comb line of the QD laser will generate two RF beat signals. The first one is the frequency detuning between the TNL and the m*^th^* comb line *δ*. The second one is the frequency detuning between the TNL and the (m+1)*^th^* comb line *Ω* = *f_rep_−δ*, where *f_rep_* is the 60 GHz laser repetition frequency. The beat signals *δ*, *Ω*, and *f_rep_* are then captured by a high-speed photodiode (PD). In this study, the frequency detuning *δ* is set to be 10 GHz, so the beat signal *Ω* will be found at 50 GHz. The phase difference between the m*^th^* and the (m+1)*^th^* comb lines $\Delta\phi$ can then be extracted through the following relationship [15]:

$$\Delta\phi=\phi_{m+1}-\phi_{m}-\phi_{rep} (S1)$$

where $\phi_{m+1}$, $\phi_{m}$, and $\phi_{rep}$ are the phases of these three signals, which can be directly calculated by applying a Fast Fourier Transform (FFT) to the real-time data. To improve the accuracy of the intermode phase difference measurements, the power for the RF signals must be strong enough. To do so, the RF signals are first amplified by a 30-dB RF amplifier (AMP) before they are analyzed by a high-speed real-time oscilloscope. Due to the bandwidth limit (18 GHz) of the AMP, the signals *Ω* and *f_rep_* should be firstly down-converted into the span of the AMP by using a low-noise RF local oscillator (LO) through an RF mixer (Methods in the main text). In this study, the frequency of the LO *f_LO_* is set to be 45 GHz, thus the signals *Ω* and *f_rep_* are down-converted to 5 and 15 GHz, respectively.

Figure S2a depicts the real-time spectrum for one measurement. The RF spectrum and the phase spectrum after an FFT analysis are shown in Fig. S2b and c, respectively. According to Eq. (S1), the phases for the recorded signals $\phi_{m+1}$, $\phi_{m}$, and $\phi_{rep}$ are then extracted to calculate the phase difference between the m*^th^* and the (m+1)*^th^* comb line. The spectral phase differences can be obtained by simply stepping the TNL across all comb lines of the QD laser, repeating the above procedure at each point.


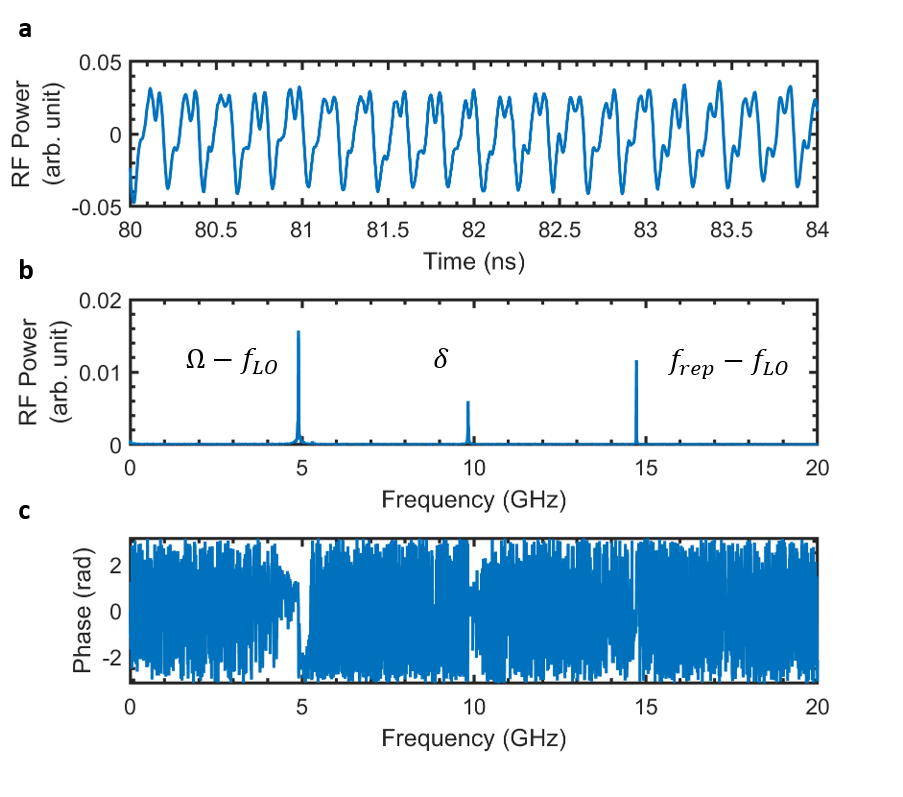


Supplementary Figure 2. **Intermode phase difference measurements in QD laser. a.** Real-time trace after amplification. **b.** RF spectrum of the real-time trace after an FFT analysis. **c.** Phase spectrum of the real-time trace after an FFT analysis.

1. **LINEWIDTH ENHANCEMENT FACTOR**

In this work, the linewidth enhancement factor ($\alpha_{H}$-factor) is measured by using the amplified spontaneous emission (ASE) method [16]. Compared to the high-speed frequency and amplitude modulation method [17], the ASE method allows for direct measurement of the differential gain and differential refractive index as a function of the carrier density in the sub-threshold operation. The differential refractive index *dn/dN* is measured by tracking the wavelength shift of the longitudinal FP mode resonances, while the differential gain *dg/dN* is extracted by tracking the variation of the net modal gain $G_{net}$. The net modal gain is measured by the FP cavity modulation depth (gain ripple) from the ASE spectra, which is calculated by using the following relationship [18]:

$$G_{net}=\frac{1}{L_{c}}\ln\left( \frac{1}{\sqrt{R_{1}R_{2}}}\frac{\sqrt{x}-1}{\sqrt{x}+1} \right) (S2)$$

where $L_{c}$ is the cavity length, $R_{1}$ and $R_{2}$ denote the power reflectivities of the front and the rear facets, respectively. $x$ accounts for the ratio of the peak-to-valley intensity levels. The differential refractive index within the active region can be calculated by the modal wavelength shift $d\lambda_{m}$ through $\frac{d\lambda_{m}}{\lambda_{m}}=\Gamma\frac{dn}{n}$, with $\lambda_{m}$and $\Gamma$ the modal wavelength and the optical confinement factor, respectively. Therefore, Eq. (S2) in the main text can be rewritten by measurable parameters such as:

$$\alpha_{H}=-\frac{4n\pi}{\lambda_{m}^{2}}\frac{{d\lambda_{m}}/{dI}}{{dG_{net}}/{dI}} (S3)$$

S
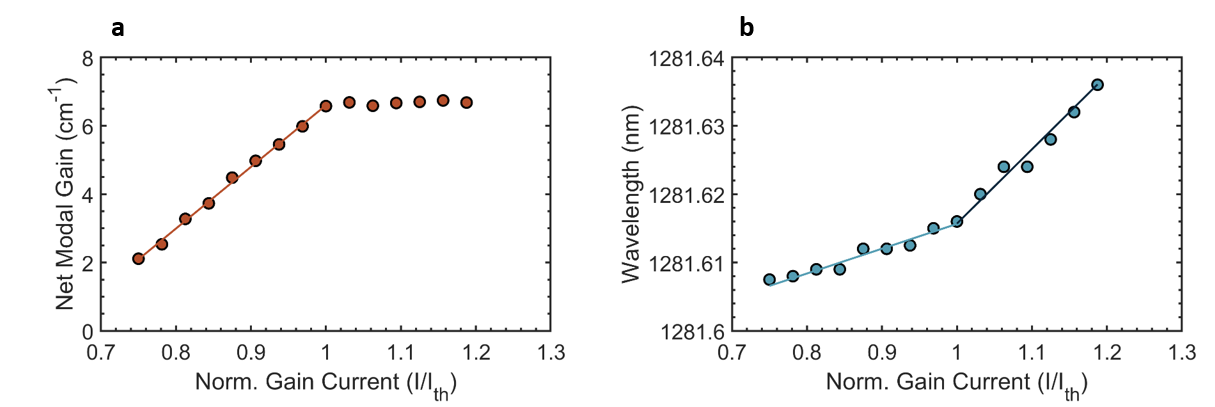


Supplementary Figure 3. **Linewidth enhancement factor measurements in QD laser. a.** Net modal gain $G_{net}$ as a function of the normalized gain current. **b.** Modal wavelength $\lambda_{m}$ as a function of the normalized gain current. The SA reverse bias is fixed to 0 V in this measurement.

Figure S3a depicts the net modal gain versus the normalized gain current for a longitudinal mode that is located at 1281.62 nm. The net modal gain increases with gain current below the threshold, until it clamps the mirror loss above the threshold. Nevertheless, it should be noted that the thermal effect also contributes to a wavelength shift, which could possibly lead to an underestimation of the $\alpha_{H}$-factor. While the wavelength shift below the threshold $d\lambda_{m,b}$ results from both the carrier-induced refractive index change and the thermal effect, the wavelength shift above the threshold $d\lambda_{m,a}$ is mainly caused by the thermal effect since the gain is clamped. The sub-threshold and the above-threshold wavelength shifts are expressed as follows:

$$d\lambda_{m,b}=\left( \frac{dn}{n}+\frac{dn_{t}}{n}+\frac{dL_{t}}{L_{t}} \right)\lambda_{m} (S4)$$

$$d\lambda_{m,a}=\left( \frac{dn_{t}}{n}+\frac{dL_{t}}{L_{t}} \right)\lambda_{m} (S5)$$

where $dn_{t}$ accounts for the refractive index change due to the thermal effect, and $dL_{t}$ denotes the thermal expansion of the laser cavity. The different modal wavelength shifts below and above the threshold are shown in Fig. S3b. Eq. (S3) can be then modified as follows:

$$\alpha_{H}=-\frac{4n\pi}{\lambda_{m}^{2}}\frac{{d(\lambda_{m,b}-\lambda_{m,a})}/{dI}}{{dG_{net}}/{dI}} (S6)$$

where the thermal effect is eliminated during the measurements of $\alpha_{H}$-factor. Nevertheless, the spatial hole burning that takes place above the threshold leads to unsaturated modal gains near the gain peak, which possibly results in an inaccurate estimation of the $\alpha_{H}$-factors for those modes. Therefore, the $\alpha_{H}$-factors for the modes near the gain peak are not performed in Fig. 4c in the main text.

1. **GROUP VELOCITY DISPERSION MEASUREMENT**

To determine the GVD of the waveguide, we need the discrete resonant frequencies of the FP cavity:

$$f_{m}=\frac{mc}{2L_{c}n_{eff}} (S7)$$

where $f_{m}=\omega_{m}/2\pi$ is the frequency of the m*^th^* longitudinal mode with the azimuthal mode number *m*. $L_{c}$ and $n_{eff}$ account for the cavity length and the frequency-dependent effective refractive index, respectively. Then, the propagation constant of the m*^th^* mode is expressed as follows:

$$\beta=\frac{2\pi n_{eff}}{\lambda_{m}}=\frac{m\pi}{L_{c}} (S8)$$

Therefore, the first-order dispersion $\beta_{1}$ and the second-order dispersion $\beta_{2}$ (GVD) can be calculated through the free spectral range (FSR) $\Delta f_{m}$ of the cavity:

$$\beta_{1}=\frac{d\beta}{d\omega_{m}}|_{m=m_{0}}=\frac{1}{2L_{c}}\frac{dm}{df_{m}}=\frac{1}{2L_{c}\cdot\Delta f_{m}} (S9)$$

$$\beta_{2}=\frac{d^{2}\beta}{d\omega_{m}^{2}}|_{m=m_{0}}=\frac{d\beta_{1}}{d\omega_{m}}=-\frac{1}{4\pi L_{c}\cdot\Delta f_{m}^{3}}\cdot\Delta\left( \Delta f_{m} \right) (S10)$$

In addition to the $\beta_{2}$, the *D*-parameter is widely used in fiber optics to analyze the GVD, which is expressed as follows:

$$D=-\frac{2\pi c}{\lambda^{2}}\beta_{2} (S11)$$

As a result, a cavity of normal dispersion shows "$\beta_{2}$>0, D<0", whereas an anomalous dispersion cavity shows "$\beta_{2}$<0, D>0".

In this work, we measure the GVD of a single-section QD FP laser whose cavity design is identical to the QD FM comb laser studied. Both facets of the QD FP laser are left as-cleaved, which allows for light couplings from both sides. The device is probed on a temperature-controlled stage for the following operations. First, the QD laser is operating above the threshold, and two AR-coated lensed single-mode fibers are used for light coupling from both facets of the laser. To measure the cold-cavity GVD, the laser is then turned off. The measurement of GVD relies on the spectral resonant frequencies of the laser cavity and the FSR. To do so, we propose to utilize a broadband light source to generate an interferometric fringe pattern from the FP cavity [19]. In this work, we use a semiconductor optical amplifier (SOA) (Thorlabs S9FC1132P) with a center wavelength of 1320 nm and a 3-dB bandwidth of 40 nm. The emission of SOA enters the FP cavity from the front facet, and the generated fringe pattern is coupled out from the rear facet. The output light is then isolated before it is analyzed by an optical spectrum analyzer (OSA) with a high resolution of 20 pm (Yokogawa AQ6370C). It should be noted that the absolute frequencies of the cavity resonances are still quite noisy due to the low-Q FP cavity, which prevents us from extracting the GVD accurately. To address this issue, we propose to do data processing to improve the resolution of FSR. Depending on the mode number N in the full spectral range, the FSR of the m*^th^* mode $\Delta f_{m}$ is calculated through the following equation:

$$\Delta f_{m}=\frac{f_{m+\frac{N}{4}}-f_{m-\frac{N}{4}}}{\frac{N}{2}-1} (S12)$$

With

$$1+\frac{N}{4}\leq m\leq\frac{3N}{4} (S13)$$

By taking advantage of a broadband light source, the resolution of FSR can be improved by a factor of $\frac{N}{2}-1$,which allows for a high-accuracy GVD measurement.

1. **GROUP VELOCITY DISPERSION CALCULATION**

The group velocity dispersion of the QD FP laser has been simulated with finite-element methods (COMSOL Multiphysics). The $n_{eff}$ of the laser over a wide range of wavelength is obtained by simulating the mode profile of the gain-section waveguide cross-section, which is then converted to $\beta_{2}$ using Eq. (S10). Apart from material dispersions, the overall TE mode dispersion is contributed mainly by the vertical mode confinement provided by the cladding. The ridge width of the waveguide has been found to not significantly change the dispersion of the laser.

1. **FOUR-WAVE MIXING IN QD LASER**

Depending on the frequency detuning between the probe and the drive frequencies, the FWM is determined by the carrier density pulsation (CDP), the carrier heating (CH), and the spectral hole burning (SHB). The effectiveness of CDP grating is determined by the interband recombination and generation rates of carriers. It has been reported that the interband ground-state carrier recovery lifetime in QD is less than 200 ps [20], which allows for an efficient CDP grating over gigahertz. In general, the CDP grating still plays a dominant role in FWM where the frequency detuning is larger than 100 GHz. The contribution of carrier heating to the FWM originates from the fact that the hot carriers must cool down to the lattice temperature through carrier-phonon interactions. In the case of InAs QD grown on GaAs, however, the CH gratings are negligible due to the discrete energy levels [21]. The SHB refers to the temporal spectral hole generated by a strong pump, in the presence of a depletion of the resonant carriers. As such, the SHB gratings are determined by the intersubband and inter-dot carrier-carrier and carrier-phonon-scattering rates. Even though the inter-dot processes are relatively slow due to the carrier capture and relaxation from the wetting layer, the intersubband processes such as the excited state to ground state relaxation are much faster. In general, the time for the intersubband process ranges from 100 to 250 fs when the wetting layer and the higher excited states are filled up by a strong pump [21, 22], which gives rise to a large SHB grating over 1 THz. As explained in the main text, the FWM bandwidth of the QD FM comb laser is as large as 1.7 THz, which results from the ultrafast carrier dynamics in the QD active region.


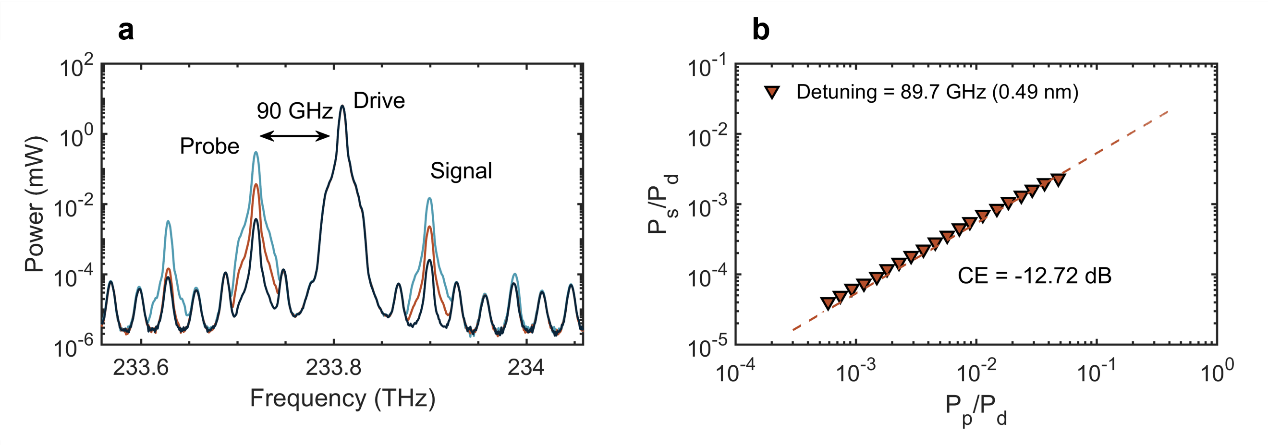


Supplementary Figure 4. **Four-wave mixing measurements in QD laser. a.** Optical spectrum in four-wave mixing (FWM) experiment, where the drive, probe, and signal frequencies are marked. The frequency detuning is 90 GHz. The plots in different colors represent different input powers of the probe laser. **b.** Signal-drive power ratio versus probe-drive power ratio when the frequency detuning is fixed to 90 GHz. The conversion efficiency (CE) is -12.72 dB in this condition. The QD laser operates with 60 mA applied to the gain and -3.3 V applied to the SA.

Figure S4a depicts the FWM spectrum when the QD laser operates with 60 mA applied to the gain and -3.3 V applied to the SA. To realize a stable injection-locking in the pump-probe experiment, the optical signal-to-noise ratio (OSNR) of the drive laser should be higher than 30 dB. It should be noted that the phase and the polarization of the drive and probe laser must be well adjusted to maximize the FWM in a semiconductor laser. As such, one needs to finely tune the probe frequency coincident with the peaks of the cavity resonances. The frequency detuning between the probe and the drive frequencies is then a multiple of the cavity FSR. The powers of the drive, probe, and signal are directly read from the optical spectrum. By finely tuning the power of the probe laser through a variable optical attenuator, the power of the converted signal is also changed. Figure S4b depicts the signal-drive power ratio as a function of the probe-drive power ratio where the frequency detuning is 90 GHz. A linear curve fit allows us to extract the conversion efficiency (CE) accurately. The FWM CEs as a function of frequency detuning can be obtained by stepping the tunable probe laser across all modes of the QD laser, repeating the above procedure at each point.

In this study, it should be noted that the maximum gain current of the QD laser for the FWM operation is limited by the power of the drive laser (10 dBm). When the QD laser operates at a gain current higher than 60 mA, it can no longer be injection-locked by the drive laser.

1. **OVERLAP FACTORS IN COLLIDING-PULSE MODE-LOCKED LASER**

Because of the colliding-pulse structure of the QD laser studied, the distribution of spectral modal gains is dependent on the position of SA inside the laser cavity. To investigate the impact of cavity geometry, a conception of overlap factors was proposed in Ref. [23]. The overlap factors for the gain and the SA, $\varepsilon_{m}^{(g)}$ and $\varepsilon_{m}^{(a)}$, respectively, are used to characterize the spatial overlap between the two interacting modes, k*^th^* and (k + m)*^th^*, and the net modal gain. The expression for these factors is written as follows:

$$\varepsilon_{m}^{(g,a)}=\int_{g,a} (u_{k}(z)u_{k+m}^{*}(z))\cos\left( \frac{m\pi}{L_{c}}\left( z+\frac{L_{c}}{2} \right) \right)dz (S14)$$

with $u_{k}$ the wave function of the laser cavity which is defined as follows:

$$u_{k}\left( z \right)=\frac{r_{L}}{N}\exp\left( -i\int_{-{L_{c}}/2}^{z} q_{k,l}\left( z^{'} \right)dz^{'} \right)+\frac{1}{N}\exp\left( i\int_{-{L_{c}}/2}^{z} q_{k,l}\left( z^{'} \right)dz^{'} \right) (S15)$$

with

$$q_{k,l}\left( z \right)=\frac{k\pi}{L_{c}}+i\gamma_{l}\left( z \right) (S16)$$

denotes the complex mode vector for the l*^th^* section of the cavity, with $\gamma_{l}=g$ and $\gamma_{l}=-a$ the gain and the absorption coefficient for the gain and the SA sections, respectively. The normalization factor *N* is then determined after the following equation is satisfied:

$$\int_{-{L_{c}}/2}^{{L_{c}}/2} \left( u_{k}\left( z \right)u_{k}^{*}\left( z \right) \right)dz=1 (S17)$$

It should be noted that the spatially averaged product of two modes is only determined by the frequency interval between them *m*×FSR but not by their absolute spectral positions. On the other hand, Eq. (S14) is a quantitative expression for the modal gain couplings since the FP cavity for the QD laser is not an idealized closed cavity. The overlap factors for the m*^th^* mode that is separated from the k*^th^* mode (peak mode) can be then calculated by using Eqs. (S14)-(S17), in which the integration is over each section of the laser cavity. For the second harmonic colliding-pulse QD laser, the integration intervals for the gain and the SA section are expressed as follows:

$$z\in\left\{ \begin{aligned} \left[ -{L_{c}}/2, {{-L}_{a}}/2 \right] (gain) \\ \left[ -{L_{a}}/2, {L_{a}}/2 \right] (SA) \\ \left[ {L_{a}}/2, {L_{c}}/2 \right] (gain) \end{aligned} (S18) \right.$$

where the zero position of *z* is assumed to be the center of the cavity, and $L_{a}$ is the length of the SA section. In this study, the parameters are set to be: $L_{c}$ = 1.35 mm, $g$ = 10 cm^−1^, $a$ = 40 cm^−1^, and the SA section length ratio $r_{SA}={L_{a}}/{L_{c}}$ = 7.8%.


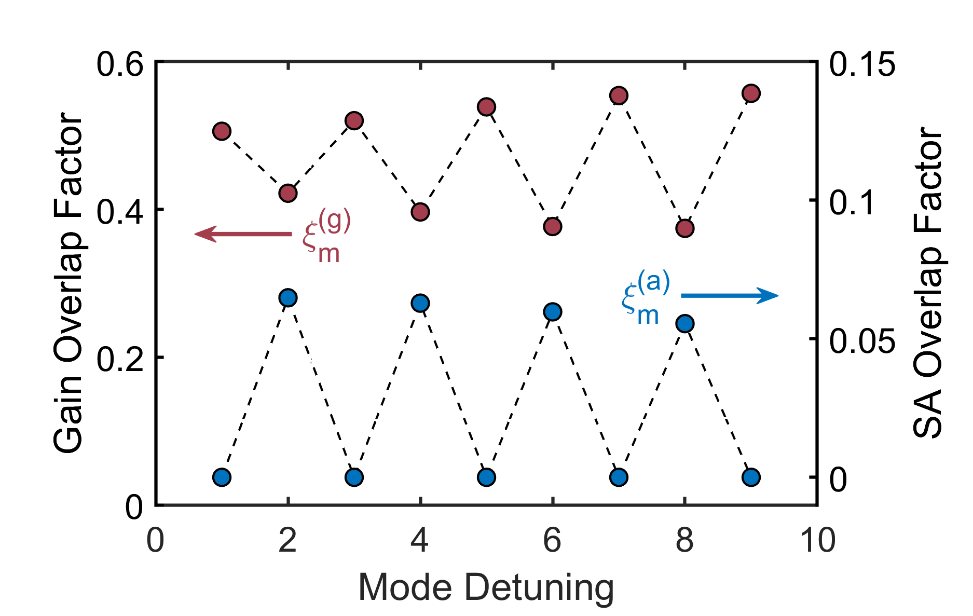


Supplementary Figure 5. **Overlap factors of the QD laser.** Overlap factors for the gain $\varepsilon_{m}^{(g)}$ (red) and the SA $\varepsilon_{m}^{(a)}$ (green) versus the number of mode intervals m for a 1.35 mm-long laser with the SA placed at the center of the laser cavity.

Figure S5 depicts the overlap factors as a function of the mode detuning number m for the gain $\varepsilon_{m}^{(g)}$ (red) and the SA $\varepsilon_{m}^{(a)}$ (green). When the QD laser operates above the threshold where the second harmonic mode-locking is developed, the SA overlap factors for the modes that are separated by an odd number of FSR from the primary mode are close to zero, which leads to a suppression of their net modal gains. As a consequence, the mode spacing of the QD laser is doubled to 60 GHz. It should be noted that a zero SA overlap factor is only valid for a closed cavity. For an FP cavity whose facet reflectivities are lower than 1, the SA overlap factor is always positive. Despite the fact that the SA overlap factors for the odd number separated modes are close to zero, the gain overlap factors for those modes are larger than the even number spaced modes. In FWM operation where the QD laser is injection locked by the drive laser, the effect of the SA overlap factor is eliminated. As a result of the larger gain overlap factors, the gains for the odd number spaced modes are larger than the even number spaced modes, the converted signals are then amplified which results in their higher conversion efficiencies.

**Reference**

[1] Z. Wang, K. Van Gasse, V. Moskalenko, S. Latkowski, E. Bente, B. Kuyken, and G. Roelkens, A III-V-on-Si ultra-dense comb laser, Light: Science & Applications 6, e16260 (2017).
[2] V. Corral, R. Guzmán, C. Gordón, X. Leijtens, and G. Carpintero, Optical frequency comb generator based on a monolithically integrated passive mode-locked ring laser with a Mach–Zehnder interferometer, Optics letters 41, 1937 (2016).
[3] M. L. Davenport, S. Skendžić, N. Volet, J. C. Hulme, M. J. Heck, and J. E. Bowers, Heterogeneous silicon/III–V semiconductor optical amplifiers, IEEE Journal of Selected Topics in Quantum Electronics 22, 78 (2016).
[4] J. H. Marsh and L. Hou, Mode-locked laser diodes and their monolithic integration, IEEE Journal of Selected Topics in Quantum Electronics 23, 1 (2017).
[5] Z. Lu, J. Liu, S. Raymond, P. Poole, P. Barrios, and D. Poitras, 312-fs pulse generation from a passive C-band InAs/InP quantum dot mode-locked laser, Optics Express 16, 10835 (2008).
[6] Z. Lu, J. Liu, P. Poole, Z. Jiao, P. Barrios, D. Poitras, J. Caballero, and X. Zhang, Ultra-high repetition rate InAs/InP quantum dot mode-locked lasers, Optics Communications 284, 2323 (2011).
[7] Z. Lu, J. Liu, P. J. Poole, Y. Mao, J. Weber, G. Liu, and P. Barrios, InAs/InP quantum dash semiconductor coherent comb lasers and their applications in optical networks, Journal of Lightwave Technology 39, 3751 (2021).
[8] T. Verolet, G. Aubin, Y. Lin, C. Browning, K. Merghem, F. Lelarge, C. Calo, A. Delmade, K. Mekhazni, E. Giacoumidis, et al., Mode locked laser phase noise reduction under optical feedback for coherent DWDM communication, Journal of Lightwave Technology 38, 5708 (2020).
[9] S. Liu, D. Jung, J. Norman, M. Kennedy, A. Gossard, and J. Bowers, 490 fs pulse generation from passively mode-locked single section quantum dot laser directly grown on on-axis GaP/Si, Electronics Letters 54, 432 (2018).
[10] S. Liu, X. Wu, D. Jung, J. C. Norman, M. Kennedy, H. K. Tsang, A. C. Gossard, and J. E. Bowers, High-channel-count 20 GHz passively mode-locked quantum dot laser directly grown on Si with 4.1 Tbit/s transmission capacity, Optica 6, 128 (2019).
[11] S. Pan, J. Huang, Z. Zhou, Z. Liu, L. Ponnampalam, Z. Liu, M. Tang, M.-C. Lo, Z. Cao, K. Nishi, et al., Quantum dot mode-locked frequency comb with ultra-stable 25.5 GHz spacing between 20° C and 120° C, Photonics Research 8, 1937 (2020).
[12] J.-Z. Huang, Z.-T. Ji, J.-J. Chen, W.-Q. Wei, J.-L. Qin, Z.-H. Wang, Z.-Y. Li, T. Wang, X. Xiao, and J.-J. Zhang, Ultra-broadband flat-top quantum dot comb lasers, Photonics Research 10, 1308 (2022).
[13] G. Kurczveil, C. Zhang, A. Descos, D. Liang, M. Fiorentino, and R. Beausoleil, On-chip hybrid silicon quantum dot comb laser with 14 error-free channels, in 2018 IEEE International Semiconductor Laser Conference (ISLC) (IEEE, 2018) pp. 1–2.
[14] D. Liang, S. Srinivasan, G. Kurczveil, B. Tossoun, S. Cheung, Y. Yuan, A. Descos, Y. Hu, Z. Huang, P. Sun, et al., An energy-efficient and bandwidth-scalable DWDM heterogeneous silicon photonics integration platform, IEEE Journal of Selected Topics in Quantum Electronics 28, 1 (2022).

[15] D. Reid, S. Murdoch, and L. Barry, Stepped-heterodyne optical complex spectrum analyzer, Optics Express 18, 19724 (2010).

[16] M. Osinski and J. Buus, Linewidth Broadening Factor in Semiconductor Lasers–An Overview, IEEE Journal of Quantum Electronics 23, 9 (1987).

[17] S. Ding, B. Dong, H. Huang, J. Bowers, and F. Grillot, Spectral dispersion of the linewidth enhancement factor and four wave mixing conversion efficiency oSf an InAs/GaAs multimode quantum dot laser, Applied Physics Letters 120, 081105 (2022).

[18] D. Bossert and D. Gallant, Improved method for gain/index measurements of semiconductor lasers, Electronics Letters 32, 338 (1996).

[19] J. Y. Lee and D. Y. Kim, Versatile chromatic dispersion measurement of a single mode fiber using spectral white light interferometry, Optics Express 14, 11608 (2006).

[20] J. Siegert, S. Marcinkevičius, and Q. X. Zhao, Carrier dynamics in modulation-doped InAs/GaAs quantum dots, Physical Review B 72, 085316 (2005).

[21] P. Borri, et al., Spectral hole-burning and carrier-heating dynamics in InGaAs quantum-dot amplifiers, IEEE Journal of Selected Topics in Quantum Electronics 6, 544 (2000).

[22] S. Schneider, et al., Excited-state gain dynamics in InGaAs quantum-dot amplifiers, IEEE Photonics Technology Letters 17, 2014 (2005).

[23] J. F. Martins-Filho, E. A. Avrutin, C. Ironside, and J. Roberts, Monolithic multiple colliding pulse mode-locked quantumwell lasers, experiment and theory, IEEE Journal of selected topics in Quantum Electronics 1, 539 (1995).
